# A Narrative Review of the Evidence for Transcatheter Aortic Valve Implants

**DOI:** 10.3390/jcdd12040113

**Published:** 2025-03-24

**Authors:** Leonard Lee, Brendan Min-Wei Chan, Melinda Spencer, Jovi Leung, Danny Liew, Hansoo Kim

**Affiliations:** 1 School of Medicine and Dentistry, Griffith University, Gold Coast, QLD 4215, Australia; 2 Faculty of Medicine and Health, University of Sydney, Sydney, NSW 2050, Australia; 3 Faculty of Medicine, University of Queensland, Brisbane, QLD 4072, Australia; 4 Faculty of Health, Medicine and Behavioural Sciences, University of Queensland, Brisbane, QLD 4072, Australia

**Keywords:** review, transcatheter aortic valve replacement, aortic stenosis, reimbursement

## Abstract

Currently, multiple transcatheter aortic valves exist in clinical use, with varying efficacy and safety rates. This review aims to evaluate the evidence base for current transcatheter valves used in the management of aortic stenosis in Australia to improve informed decision making for both clinicians and patients. The evidence base included published peer-reviewed human studies of aortic valves with approval for use in Australia through the Therapeutic Goods Administration (TGA). Embase was utilised on the 17 September 2024, and one hundred ninety-seven publications met the inclusion criteria, including six from citation searching. The Sapien series led with the largest number of patients reported in the literature (*n* = 91,614) and publications (*n* = 147), followed by the CoreValve/Evolut series with 65,459 patients and 125 publications. Evidence for both of these transcatheter aortic valve series were also published in high impact journals, with the greatest H-index journal being The New England Journal of Medicine. In conclusion, the evidence base for the safety and efficacy of the Sapien and CoreValve/Evolut series currently leads in both quantity and quality. This review also summarises the Australian medical device regulatory and funding process in the context of transcatheter aortic valves.

## 1. Background

Aortic stenosis (AS) is among the most common of valvular heart diseases, with an overall prevalence of 1.48% among Australians aged over 54 years, affecting an estimated minimum of 97,000 Australians [1]. Left untreated, severe AS results in symptoms such as angina pectoris, syncope, and dyspnoea and is associated with a one-year mortality of 30–50% [2]. Affected patients have significantly reduced quality of life (QoL), with 32.5 disability-adjusted life years (DALYs) lost per 1000 men and 57.5 DALYs lost per 1000 women [3]. The societal cost of premature mortality in Australia from AS in 2017 was estimated to be AUD 629 million among men and AUD 735 million among women. However, given the current ageing population, and the association of AS with age, the burden of disease will likely continue to increase [3]. The mortality and morbidity burden of AS, together with its increasing prevalence, necessitates cost-effective treatment options [1].

Traditionally, AS patients were treated with surgical aortic valve replacement (SAVR), but for those with a high risk of post-operative mortality, categorised by Society of Thoracic Surgeon Predicted Risk of Mortality (STS PROM) scores, SAVR represented a poot treatment option [2,4]. In its place, transcatheter aortic valve replacement (TAVR) revolutionised care for high-risk patients. Over the past ten years, TAVR has been shown to be cost-effective compared to SAVR among patients with intermediate and low risk as well [4]. Since 2021, the Australian government has subsidised TAVR for patients with severe symptomatic AS, regardless of the risk of surgical mortality [5]. Compared to SAVR, TAVR is associated with fewer procedural complications such as myocardial infraction, aortic rupture, and haemorrhage, as well as a shorter hospital stay [6,7].

The main types of TAVRs comprise self-expanding valves (SEVs), balloon-expandable valves (BEVs), and mechanically expandable valves (MEVs) [8]. MEVs were recalled due to delivery system issues, and currently none are registered in the US, Europe, China, India, and Australia [8]. Irrespective of type, valves are made of organic tissue and mounted inside a metallic frame, with the differentiator being the mechanism by which the valve expands once implanted [9].

In Australia, regulatory approval for TAVR valves is provided by the Therapeutic Goods Administration (TGA) and requires listing on the Australian Register of Therapeutic Goods (ARTG) before usage [10]. The TGA regulates all medical interventions including medications and medical devices and stratifies approval requirements based on risk scores formulated from the invasiveness of the device, duration of device implantation, location of implant, and device indications. Prosthetic heart valves are categorised under the highest risk level (high risk, Class III). For Class III medical devices, approval includes the conformity assessment certification, in which the device manufacture and design are certified by the TGA or a European Notified body, a Clinical Evaluation Report and Instructions for Use, and Premarket Approval, which involves rigorous standards involving clinical evidence [10]. Currently, the TAVR valves available in Australia include the Sapien 3 and Sapien 3 Ultra (Edwards Lifesciences, Irvine, CA, USA); Evolut R, Evolut R+, Evolut PRO, and Evolut FX (Medtronic, Minneapolis, MN, USA); and the Portico and Navitor (Abbott, Chicago, IL, USA). FDA-approved valves also include the Medtronic CoreValve line, Boston Scientific Acurate Neo valves, Abbott Portico and Navigator valves, and the Edwards LifeSciences Sapien valves [8].

Public funding for registered TAVR valves in Australia is overseen by the Australian Medical Services Advisory Committee (MSAC) [11]. This process is underpinned by four main stages. The first is the pre-assessment phase, during which applicants are made aware of the evidence required for approval, followed by the PICO (population, intervention, comparator, outcomes) confirmation stage in which a health technology assessment group is selected to develop the PICO framework for an economic evaluation. Next, the application assessment phase results in a report detailing the clinical and economic evidence, including a consumer impact statement. This is submitted for MSAC consideration. Finally, in the appraisal phase, the MSAC formally considers if the intervention should be listed on the Medical Benefits Schedule (MBS). Successful applicants can then apply for listing of the product on the Prostheses List, which tables medical devices for which health insurers must pay a benefit to patients who have appropriate coverage [11].

Continuous improvements in valve design across the valve categories, as well as new market entrants from multiple device manufacturers, have resulted in a multitude of valve options for use in TAVRs. The current pricing in the Prostheses List for all TAVR valves is AUD 22,932, irrespective of the valve type and quality of evidence supporting its efficacy [12]. Many studies comparing different valve types and brands have been published internationally, varying in quality of evidence and study design. The present review aims to assess the evidence base for current valves used in the management of AS in Australia.

## 2. Methods

A systematic scoping review of published journals for aortic stenosis patients was conducted on 17 September 2024 using Embase. Terms related to TAVR or implantation, aortic stenosis, and clinical trials were utilised. Additionally, currently available valve brand names were obtained from the publication by Vinayak et al. (2024) and added to the search strategy [8]. The search was additionally restricted to aortic stenosis (excluding other valve diseases) and primary TAVRs (excluding ‘valve in valve’ or revision TAVRs). In accordance with the Preferred Reporting Items for Systematic Reviews and Meta-Analyses extension for scoping reviews (PRISMA-ScR), the final included papers were independently reviewed by two authors (BC and LL) [13]. The papers included were required to be full text accessible, in English, and involving patients with aortic stenosis undergoing primary TAVR. Studies also required specific stratification of outcomes by valve type. Additionally, mechanically expandable valves were excluded due to delivery system issues [8]. Papers were excluded if no primary data were collected by the study authors (i.e., papers comparing two primary cohorts) due to the potential of bias resulting from the outcomes of both primary studies being known before comparison. Furthermore, studies comparing primary trial data with the trial data from another primary paper were excluded if no patient matching occurred (either clinically, such as with the STS risk score, or statistically, such as via propensity matching). Finally, studies with outcomes unrelated to valve performance, such as imaging techniques or intraoperative outcomes, were excluded. The full search strategy is accessible in Appendix A.1.

The secondary aim of our paper was to summarise the current approved indications for valves listed on the TGA. This was undertaken by searching the ARTG. Furthermore, to evaluate the quality of evidence for the included TAVR valves, a quality assessment table aligned with MSAC guidelines was constructed, which categorised evidence as high quality (prospective randomised controlled trials), moderate quality (prospective clinical trials with propensity matched cohorts), low quality (retrospective single-arm observational studies with propensity matching), and very low quality (single-arm observational studies with no propensity matching). Additionally, we evaluated and summarised the top 10 journals in which evidence was published and the journal quartile and H-index, which were obtained through the Scimago Journal and Country Rank [14].

## 3. Results

The search yielded 197 studies for inclusion, from an original 3068 papers, with the reasons for exclusion explained in Supplementary S1—PRISMA diagram.

**Figure 1:**
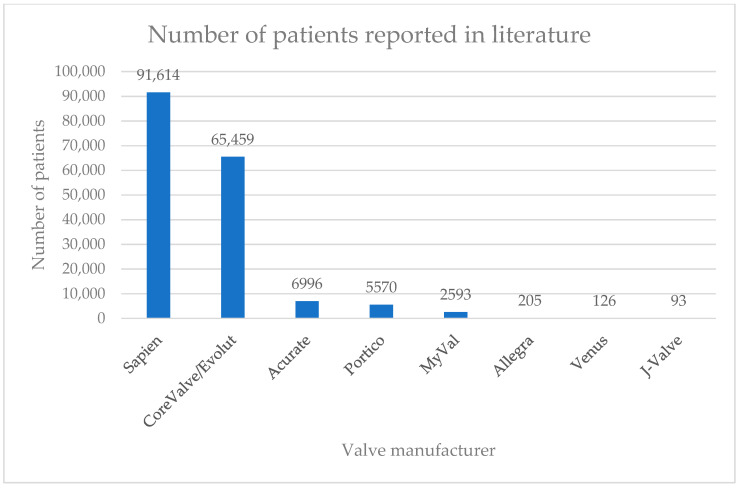
Number of patients reported in the literature by valve manufacturer.

FDA-approved valves grouped into their respective manufacturer categories were involved in most of the included studies, with only five observational studies published for all non-FDA approved valves. Of the 197 included studies, a Sapien valve was evaluated in 147 studies, a CoreValve/Evolut in 125, an Acurate in 26, and a Portico in 17, with the respective patient numbers listed in Figure 1. Overall, Sapien and CoreValve/Evolut were studied in the largest cohorts.

The numbers of studies, stratified by valve type and manufacturer, are summarised in Table 1.

For BEVs, the findings were aligned with the duration of market participation, with Sapien valves (Edwards Lifesciences, Irvine, CA, USA) having a greater number of studies compared to other valve manufacturers. Other potential market entrants, such as the MyVal series from Meril (Vapi, Gujarat, India) and domestically the DurAVR from Anteris Technologies (Brisbane, Australia), had comparatively fewer studies that fulfilled the inclusion criteria of our review.

Within the SEV category, the Evolut series (Medtronic, Minneapolis, MN, USA), the Accurate Neo series (Boston Scientific, Marlborough, MA, USA), and the Portico (Abbott, Chicago, IL, USA) had the greatest number of studies. Newer valve manufacturers in this category also had comparatively fewer studies fulfilling our inclusion criteria. Given the inclusion criteria requiring studies comparing a TAVR and a second intervention, which often was another TAVR, the summed total studies for each valve exceeded the total included studies. Nonetheless, Sapien 3 (*n* = 65), CoreValve (*n* = 94), and Acurate Neo (*n* = 25) had the greatest number of studies for each respective valve series. A table detailing the frequency of each valve type within the studies that matched our review inclusion criteria divided by observational and randomised controlled trial is listed in Appendix A.2.

Regarding journal quality, Sapien and CoreValve/Evolut had at least one publication in The New England Journal of Medicine (quartile 1, H-index 1184), while Acurate and Portico valves each had at least one publication in the Lancet (quartile 1, H-index 895) and Circulation (quartile 1, H-index 674). A complete list of the top 10 highest impact journals, stratified by valve manufacturer is listed in Appendix A.3.

With respects to currently approved TAVR valves in Australia, the search of the ARTG found the Sapien, Evolut, Portico, and Navitor series at various approval dates for different indications. A summarisation of valve type and their approval date for each indication is available in Table 2.

**Figure 2:**
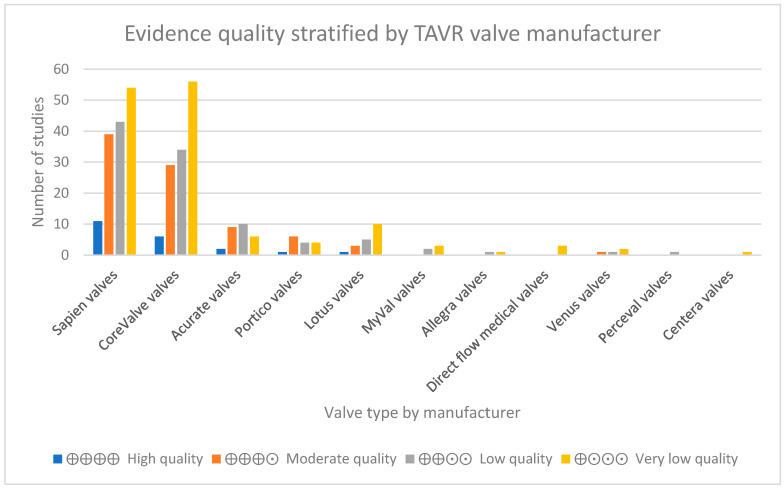
Evidence quality stratified by TAVR valve manufacturer.

The valve type associated with the highest number of high-quality randomised controlled trials (RCTs) as a proportion of its total evidence base was Sapien, with three high quality studies, followed by CoreValve and Acurate at two, and Portico with one, with all others not being studied in RCTs. Refer to Figure 2 for a representation of the evidence quality stratified by manufacturer.

## 4. Discussion

The results of our scoping review demonstrate differences in the quality of evidence associated with TGA-approved TAVR valves in Australia. Sapien, CoreValve, and Portico valves are supported by five or more moderate-quality papers and one or more high-quality papers. The next ranked valve was Lotus valves, which were supported by six moderate- and one high-quality study. Notably, four recently published, high-quality randomised controlled trials involving Sapien (*n* = 3), CoreValve/Evolut (*n* = 3), Acurate (*n* = 1), and Portico (*n* = 1) were excluded as not all of the participants per valve type were extractable (e.g., Sapien 3 and Sapien 3 Ultra were described together) [15,16,17,18].

In China, which favours the use of domestically produced products, four SEVs and one BEV valve are currently available [19]. All four SEVs are domestic, including the J-Valve (JieCheng Medical) [8,19]. The BEV is Edwards LifeSciences’ Sapien [19]. The four SEVs do not reach the evidence threshold required for TGA approval in Australia. However, TAVR has only recently become widely available in China, with the first Venus A-Valve approved for use in 2017, suggesting future adaptations and opportunities for clinical trials [20]. Another market that has recently adopted TAVR for severe aortic stenosis patients at risk of surgical mortality is India. While internationally designed valve types such as Evolut R, Sapien 3, and CoreValve have been used in studies in the Indian market, domestic designs are also concurrently being developed [21]. One such entrant is the MyVal and MyVal Octacor from Meril Life, a BEV with a value proposition arising from its wide range of sizes, from 20 mm to 32 mm [8]. Mismatches between valve size and aortic annulus can lead to paravalvular leakage and higher transvalvular gradients [22]. However, device equivalence or superiority across Valve Academic Research Consortium (VARC) outcomes against current market leaders remain uncertain, with no head-to-head RCTs comparing MyVal to Sapien, CoreValve, Acurate, and Portico.

Notably, the evidence base for the original CoreValve and Sapien, for which all subsequent generations of their respective valves were modelled from, extend beyond this review and include early phase I for safety and efficacy studies with no comparator [23,24,25,26,27,28,29,30,31,32,33,34,35,36,37,38,39,40,41]. These studies represent a development program similar to the traditional clinical phases (I to III) present in the rigorous process for medication development, and further increases the confidence in the evidence base of these valves [42].

Currently, all TAVR valves on the Prostheses List are priced equally, suggesting an equivalence among them. However, our paper demonstrates differences in the strength of the evidence supporting the valves. This suggests a need for a more rigorous process for listing devices on the Prostheses List, such as with the listing of pharmaceutical agents on the Pharmaceutical Benefits Scheme (PBS) [43]

## 5. Conclusions and Future Directions

TAVR has been a revolutionary advancement for the treatment of AS. There are distinct valve types, supported by varying evidence bases. Currently, the Sapien and Evolut series of valves have the largest and highest quality of evidence to support their efficacy and safety. The present review has also summarised the evidence base for every valve registered for use in the Australian market, which will assist with informed decision making. Future investigations in this area should aim to evaluate TAVR technologies through high-quality, randomised controlled trials, particularly for emerging manufacturers or new valve types to establish clinical equivalence or superiority to clinically efficacious options with regulatory approval. Further, the current identical pricing on the Prostheses List in Australia implies clinical equivalence between all approved valves, and it may be appropriate either for manufacturers to meet standardised evidence requirements for certain reimbursement prices (i.e., reduced reimbursement for reduced clinical efficacy confidence) or for clinicians and patients to be made aware of the different options and their supporting evidence.

## Figures and Tables

**Table 1:** Number of studies for valves found in review stratified by manufacturer and generation.

**Self-Expandable Valves**						
Medtronic, Minneapolis, MN, USA						
Number of studies	CoreValve	Evolut R		Evolut PRO	Evolut PRO+	Evolut FX
	94	43		23	4	1
Boston Scientific, Marlborough, MA, USA						
Number of studies	Acurate Neo	Acurate Neo 2				
	27	4				
Abbott, Chicago, IL, USA						
Number of studies	Portico	Navitor				
	11	0				
New Valve Technology, Hechingen, Germany						
Number of studies	Allegra					
	1					
JC Medical Inc., Burlingame, CA, USA						
Number of studies	J-valve					
	5					
Venus Medtech Inc., Hangzhou, China						
Number of studies	Venus-A	Venus-A-Plus	Venus-A-Pro			
	4	0	0			
**Balloon-expandable valves**						
Edwards Lifesciences, Irvine, CA, USA						
Number of studies	Sapien	Sapien XT	Sapien 3	Sapien 3 Ultra	Sapien 3 Ultra Resilia	Sapien X4
	64	44	65	6	1	0
Meril, Vapi, Gujarat, India						
	MyVal	MyVal Octacor				
Number of studies	5	0				
Anteris Technologies, Toowong, Australia						
	DurAVR					
Number of studies	0					

**Table 2:** TGA-approved TAVR valves in Australia, their indication, and approval date.

Indication	Valve Type (Approval Date)
Symptomatic severe aortic stenosis (all risk categories)	**Sapien** 3 (26 March 2019), Sapien 3 Ultra (18 September 2020), **Evolut** R (9 July 2020), Evolut R+ (31 October 2019), Evolut PRO (14 July 2020), Evolut FX (7 December 2023)
Symptomatic severe aortic stenosis at **high risk only**	**Portico** (4 January 2017), **Navitor** (several different models: 23 November 2022, 21 May 2024, 6 June 2024)
Symptomatic severe aortic stenosis at **extreme risk only**	**Navitor** (several different models: 23 November 2022, 21 May 2024, 6 June 2024)
Valve replacement (valve-in-valve, secondary)	**Sapien** 3 (26 March 2019), Sapien 3 Ultra (18 September 2020)
Valve replacement (surgical, secondary)	**Sapien** 3 (26 March 2019), Sapien 3 Ultra (18 September 2020), **Evolut** R (9 July 2020), Evolut R+ (31 October 2019), Evolut PRO (14 July 2020), Evolut FX (7 December 2023)

## Data Availability

All data generated or analysed during this study have been included in this published article and its Supplementary Materials. No additional data were used. The authors are contactable for additional clarifications related to the data.

## Supplementary Materials

The following supporting information can be downloaded at: https://www.mdpi.com/article/10.3390/jcdd12040113/s1. Supplementary S1. PRISMA Diagram.

## Appendix A

### Appendix A.1. Search Strategy 1

(((((‘transcatheter aortic valve replacement’/exp OR ‘transcatheter aortic valve replacement’ OR ((‘transcatheter’/exp OR transcatheter) AND aortic AND (‘valve’/exp OR valve) AND (‘replacement’/exp OR replacement)) OR ‘transcatheter aortic valve implantation’/exp OR ‘transcatheter aortic valve implantation’ OR ((‘transcatheter’/exp OR transcatheter) AND aortic AND (‘valve’/exp OR valve) AND (‘implantation’/exp OR implantation))) NOT mitral NOT pulmonary NOT reintervention NOT redo) AND (aortic AND stenosis NOT mitral)) AND ([2000–2024]/py NOT ’valve in valve’)) AND (evolut OR acurate OR portico OR navitor OR hydra OR allegra OR trilogy OR ‘j valve’ OR ‘venus a’ OR vita OR taurus OR sapien OR myval)) AND ((‘clinical article’/de OR ‘clinical study’/de OR ‘clinical trial’/de OR ‘clinical trial topic’/de OR ‘cohort analysis’/de OR ‘comparative effectiveness’/de OR ‘comparative study’/de OR ‘controlled clinical trial’/de OR ‘controlled study’/de OR ‘device comparison’/de OR ‘diagnostic test accuracy study’/de OR ‘evidence based medicine’/de OR ‘feasibility study’/de OR ‘human tissue’/de OR ‘intention to treat analysis’/de OR ‘intermethod comparison’/de OR ‘intervention study’/de OR ‘major clinical study’/de OR ‘medical record review’/de OR ‘model’/de OR ‘multicenter study’/de OR ‘multicenter study topic’/de OR ‘observational study’/de OR ‘open study’/de OR ‘pilot study’/de OR ‘proportional hazards model’/de OR ‘prospective study’/de OR ‘questionnaire’/de OR ‘randomized controlled trial’/de OR ‘randomized controlled trial topic’/de OR ‘retrospective study’/de OR ‘sample size’/de OR ‘simulation’/de OR ‘trend study’/de)).

### Appendix A.3. Journal Quality

**Table:**

	Top 10 Journals	Quartile	H-Index	Number of Publications
Sapien valves Q1: 101 Q2: 33 Q3: 7 Q4: 6	The New England Journal of Medicine The Lancet Circulation Journal of the American College of Cardiology European Heart Journal Scientific Reports American Journal of Cardiology Annals of Thoracic Surgery Journal of Thoracic and Cardiovascular Surgery Heart	1 1 1 1 1 1 1 1 1 1	1184 895 674 493 348 315 237 222 218 207	4 1 4 11 7 1 12 2 2 1
CoreValve valves Q1: 82 Q2: 26 Q3: 9 Q4: 6	The New England Journal of Medicine The Lancet Circulation Journal of the American College of Cardiology European Heart Journal Scientific reports American Journal of Cardiology Annals of Thoracic Surgery Journal of Thoracic and Cardiovascular Surgery Heart	1 1 1 1 1 1 1 1 1 1	1184 895 674 493 348 315 237 222 218 207	4 1 5 8 6 1 11 1 1 3
Acurate valves Q1: 18 Q2: 7 Q3: 1 Q4: 0	The Lancet Circulation European Heart Journal Circulation Scientific reports JACC: Cardiovascular Interventions International Journal of Cardiology Catheterization and Cardiovascular Interventions Circulation: Cardiovascular Interventions Journal of Clinical Medicine EuroIntervention	1 1 1 1 1 1 1 1 1 1	895 674 348 315 154 146 130 116 113 95	1 1 1 1 1 3 2 2 2 2
Perceval valves	Thoracic and Cardiovascular Surgeon	2	58	1
Centera valves	Journal of Thoracic Disease	2	88	1
Portico valves Q1: 9 Q2: 6 Q3: 0 Q4: 0	The Lancet Scientific reports American Journal of Cardiology International Journal of Cardiology Catheterization and Cardiovascular Interventions Circulation: Cardiovascular Interventions Journal of Clinical Medicine Clinical Research in Cardiology Revista Espanola de Cardiologia Journal of Invasive Cardiology	1 1 1 1 1 1 1 1 2 2	895 315 237 146 130 116 113 82 76 64	1 1 1 1 1 1 1 2 2 2
Lotus valves Q1: 10 Q2: 6 Q3: 1 Q4: 2	Circulation Journal of the American College of Cardiology Scientific reports Internal Medicine Journal International Journal of Cardiology Eurospace Internal Medicine Journal Circulation: Cardiovascular Interventions EuroIntervention Clinical Research in Cardiology	1 1 1 1 1 1 1 1 1 1	674 493 315 188 146 128 188 116 95 82	1 2 1 1 1 1 1 2 1 1
MyVal valves	International Journal of Cardiology Journal of Clinical Medicine Revista Espanola de Cardiologia	1 1 2	146 113 76	2 2 1
Allegra valves	International Journal of Cardiology Revista Espanola de Cardiologia	1 2	146 76	1 1
Direct flow medical valves	International Journal of Cardiology Journal of Cardiology Anatolian Journal of Cardiology	1 2 3	146 63 37	1 1 1
Venus valves	Catheterization and Cardiovascular Interventions Annals of Translational Medicine International Heart Journal Academic Journal of Second Military Medical University	1 1 3 4	130 75 54 11	1 1 1 1
