# Supplementary figures and images for: A Narrative Review of the Evidence for Transcatheter Aortic Valve Implants
Source: J Cardiovasc Dev Dis. 2025 Mar 24;12(4):113. doi: 10.3390/jcdd12040113 (PMC12027517; doi:10.3390/jcdd12040113)

# Supplementary 1. PRISMA Diagram

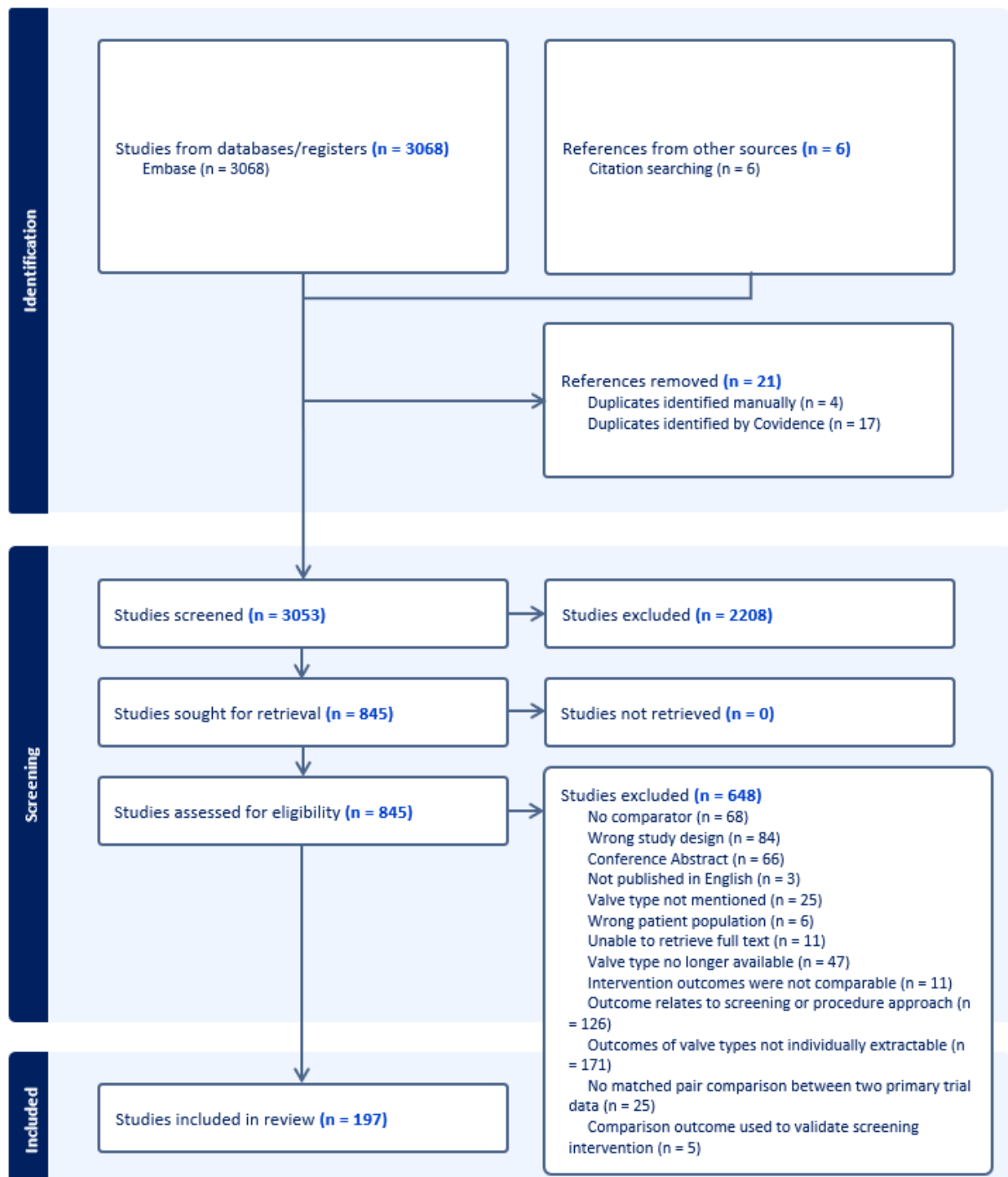

Supplement: Supplementary file 1 [file jcdd-12-00113-s001.zip › jcdd-3528385-supplementary.pdf]
